# Radiological Comparative Study Between Conventional and Nano Hydroxyapatite With Platelet-Rich Fibrin (PRF) Membranes for Their Effects on Alveolar Bone Density

**DOI:** 10.7759/cureus.32381

**Published:** 2022-12-10

**Authors:** Ali Mahfuri, Asaad Shehada, Khaldoun Darwich, Ruwaida Saima

**Affiliations:** 1 Oral and Maxillofacial Surgery, Damascus University - Faculty of Dentistry, Damascus, SYR; 2 Periodontology, Damascus University - Faculty of Dentistry, Damascus, SYR

**Keywords:** bone density, platelet rich fibrin prf, cbct, nanohydroxyapatite, bone graft, socket preservation

## Abstract

Background: The entry of the concept of nanotechnologies into the field of biomaterials has improved the results of their use in regenerative therapies based on the principles of tissue engineering, due to its improvement of the physical properties of materials manufactured in this way, so it has become possible to produce particles of hydroxyapatite in nano sizes.

Aim of the study: This study will evaluate the efficacy of applying nanohydroxyapatite paste and platelet-rich fibrin (PRF) as a barrier membrane in comparison with traditional hydroxyapatite (a powder consisting of macro-sized particles) and PRF as a barrier membrane in symmetrically extracted alveoli of the mandible.

Materials and methods: The research sample consisted of 40 lower alveoli (symmetrical) of the extracted teeth. The study samples were divided randomly into two groups. Group 1: A paste of nanohydroxyapatite with PRF as a barrier membrane was applied to one side of the extraction. Group 2: Hydroxyapatite powder with PRF as a barrier membrane was applied to the alveolus from the opposite side of Group 1 (the opposite side of the extraction). Three radiographs were performed by cone beam conventional tomography (CBCT) in three consecutive periods to conduct the radiological study (T0: immediately after extraction and grafting, T1: after three months, T2: after six months).

Results: The mean of the radiographic bone density of the hydroxyapatite powder group at time T0 is 824.36 HU with a standard deviation of ±277.29 HU, and at time T1 is 1119.93 HU with a standard deviation of ±306.93 HU, and at time T2 is 1074.14 HU with a standard deviation of ±223.62 HU, with statistically significant differences when comparing the amount of change in radiographic bone density at time T0 and T1 with P < 0.05, and at time T0 and T2 with P < 0.05, but there were no significant differences when comparing the amount of change in radiographic bone density in T1 and T2 times with P > 0.05.

The mean radiographic bone density of the nanohydroxyapatite paste group at time T0 is 629.88 HU with a standard deviation of ±193.64 HU, and at time T1 is 960.67 HU with a standard deviation of ±256.88 HU, and at time T2 is 743.87 HU with a standard deviation of ±180.96 HU, and in the time T0 and T1 with P < 0.05, and in the time T0 and T2 with P < 0.05, and in the time T1 and T2 with P < 0.05.

Statistically, significant differences have been found between bone density change T1, T2 in the nanohydroxyapatite paste group and bone density change T1, T2 in the hydroxyapatite powder group P<0.05, which expresses a greater loss of density in the nanohydroxyapatite group, and thus the resorption of the bone graft and the placement of new bone tissue.

Conclusion: Within the limits of our study, the results demonstrated that the use of traditional hydroxyapatite powder and nanohydroxyapatite paste increases the radiographic bone density, nanohydroxyapatite paste has a greater absorbency after 3 months compared with traditional hydroxyapatite powder which helps replace it by natural bone tissue.

## Introduction

Bone is described as a specialized mineralized connective tissue [1,2], which consists of a mineralized organic scaffold, mostly collagen, containing calcified intercellular materials [3], and containing many types of specialized cells [2,4,5]. It is known and certain that the alveolar process suffers from resorption after tooth extraction, and this has been proven [6]. The phenomenon of alveolar resorption and remodeling is considered a natural phenomenon that affects the alveolar bone immediately after extraction, but it is undesirable and negatively affects the placement of dental implants later [7].

Healing events in the socket of the extracted tooth reach their peak with the formation of the woven bone, which is re-released, ending with the restoration of the space in the socket. It occurs in humans during the first four weeks, which are replaced by fibrous tissue and blood vessels, followed by the differentiation of the bone tissue and the formation of lamellar bone between the fourth and eighth week after extraction, followed by the stage of bone maturity [1,8], while Boyne PJ stated that the formation of ossification in the socket begins about the tenth day after extraction [9]. The formation of new bone is the most important criterion in the success of oral surgical treatments, so the focus has been on searching for new ways to improve oral surgical treatment techniques and their effects on patients [10].

Socket preservation is described as the application of bone regeneration directed concurrently with tooth extraction to control post-extraction bone resorption [7]. Where many scientists have studied the advantages of using bone grafts in the cavities of extracted teeth, considering that these grafts affect the natural healing of the dental alveoli [11].

Hydroxyapatite has proven to be a suitable bone substitute with good results in socket preservation after extraction, as hydroxyapatite is one of the most common forms of calcium phosphate in bone regeneration applications due to its structure and composition similar to natural bone minerals, However, hydroxyapatite remains for a very long time within the bone tissue without being absorbed [12]. In addition, nanohydroxyapatite showed great similarity with the natural hydroxyapatite in terms of surface roughness, hydrophilicity, and larger surface area, which led to more positive biological effects, improved protein adsorption, mesenchymal cell adhesion, and improved differentiation and cellular proliferation, and it has a high absorbency to be replaced by bone tissue [13,14].

Also, it is difficult for cells to infiltrate into the granular form of hydroxyapatite in comparison to the paste form, which make the patient susceptible to secondary infection [15]. The PRF is routinely used as a barrier membrane to redirect the tissues and cells to re-form the periodontal tissues, which leads to the formation of mineralized tissue [16].

## Materials and methods

Study design

The research sample consisted of 40 mandibular alveoli (symmetrical) of extracted teeth from a number of patients attending the Department of Oral and Maxillofacial Surgery at the Faculty of Dentistry at Damascus University, who met the inclusion criteria in the study. The sample size was calculated By G.power3.1 (with 95% power and a 5% alpha error based on a two-sided test). Effect size (1.7) was calculated by the result of a nanohydroxyapatite pilot study which contain bone density changes between grafting time and after three months in 10 extraction sockets, eventually, the total sample size was seven samples in each group a minimal number. Bone density estimated in Hounsfield using the onDemand3D dental software. CBCT data have been analyzed by SPSS Statistics 20. The study samples were divided randomly into two groups; Group 1: A paste of nanohydroxyapatite with platelet-rich fibrin (PRF) barrier membrane was applied to one side of the extraction (N). Group 2: Hydroxyapatite powder with PRF as a barrier membrane was applied to the alveolus from the opposite side of Group 1 (the other side of the extraction) (M).

This is a comparative radiological study. The selection of the research samples was subjected to a set of conditions, which that the patient's age ranged between 18 and 60 years, the patient should have a symmetrical tooth extraction (single or multiple in the lower jaw), the indication for extraction is for restorative or endodontic causes, provided that there is no resorption in the alveolar margin, or that the existing resorption does not exceed one-third of the alveolar margin, with the exclusion of the teeth, indicated for extraction due to the causes of periodontal disease, the absence of general or local diseases that constitute a contraindication for tooth extraction, the absence of metabolic diseases that affect the normal metabolism of bone, the patient did not undergo the previous radiotherapy in the maxillofacial area, not to be subject to drug treatments that affect the normal metabolism of the bone and the female patient is not pregnant.

Working methodology

General Examination of the Patient

*I*t is a medical interrogation of the patient about its health in general and focuses on diseases that have an impact on the bone quantitatively and qualitatively and therefore on bone restoration.

Clinical Intraoral Examination

It includes examining the patient's oral cavity clinically to assess the condition of the teeth indicated for extraction, adjacent, opposite teeth, and examination of the oral mucosa and periodontal tissues.

Radiological Examination

A panoramic radiograph is used to assess the condition of the teeth to be extracted and the condition of the adjacent teeth and the surrounding alveolar bone. Obtaining the consent of the patient to undergo the research procedures.

Surgical Steps

The surgical procedure was performed by a master's degree in oral and maxillofacial surgery student. A blood sample of 40 mL was drawn from the patient and centrifuged at 3,000 rpm for 10 minutes to obtain the PRF, the patient was asked to rinse the mouth using chlorhexidine solution, and the local anesthesia was done using the local anesthetic 2% lidocaine with vasoconstrictor Adrenaline 1/80,000, bilateral extraction using appropriate tools, injecting nanohydroxyapatite paste into the socket at the site of extraction until the alveolar cavity is filled, preparing one of the PRF samples in the form of a barrier membrane and then covering the bone graft with it, placing the hydroxyapatite powder bone graft and covering it with PRF barrier membrane. Bone grafts were from Inc Fluidinova from Portugal. At the end of the extraction, suturing the gingival margins using 3/0 silk thread to hold PRF in place, and the blinding was unable for the surgeon while performing the surgery because the bone grafts have different forms, While the person who studied the CBCT images was blinded to the type of grafts used. Giving the patient post-extraction instructions.

Postoperative Procedures

Performing a CBCT image of the patient immediately after the extraction process and keep in touch with the patient and ask him to review after three and six months for a CBCT radiography. Carrying out a radiological study of the images to assess the radiological bone density after three months (T1) and after six months (T2).

The change in radiographic bone density was measured in the middle of the alveolar socket (determined from the first image at time T0), and the distance of the measurement space was fixed from the reference line, and this distance was re-applied to the following images for subsequent measurements, and the amount of change in radiographic bone density was mentioned when comparing each of the three times.

Study of the Amount of Change in the Radiographic Bone Density

The effect of the used treatment method on the change in radiographic bone density (in Hounsfield) was studied, as a density measurement was made in a square with an area of 5 mm^2^ and located in the middle of the socket.

The t-test was conducted for independent samples to study the significance of the differences in the radiographic bone density, where the amount of change was compared between the extraction group with the application of hydroxyapatite powder and the extraction group with the application of nanohydroxyapatite paste in the research sample. Additionally, the one-way repeated measures ANOVA test for each group with pairwise comparisons.

Clinical cases

First Clinical Case

**Figure 1:**
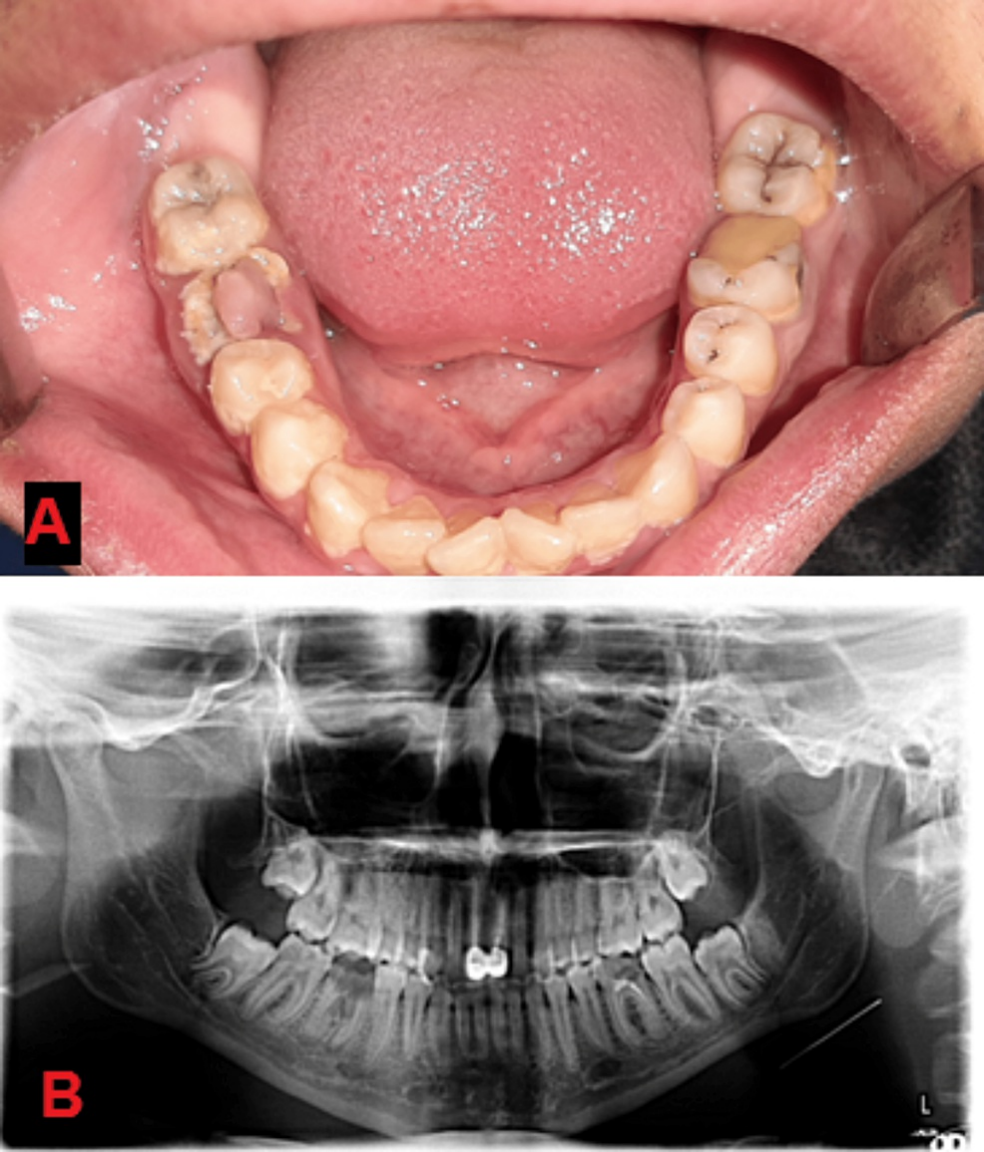
(A) Diagnostic clinical image, (B) diagnostic panoramic image.

**Figure 2:**
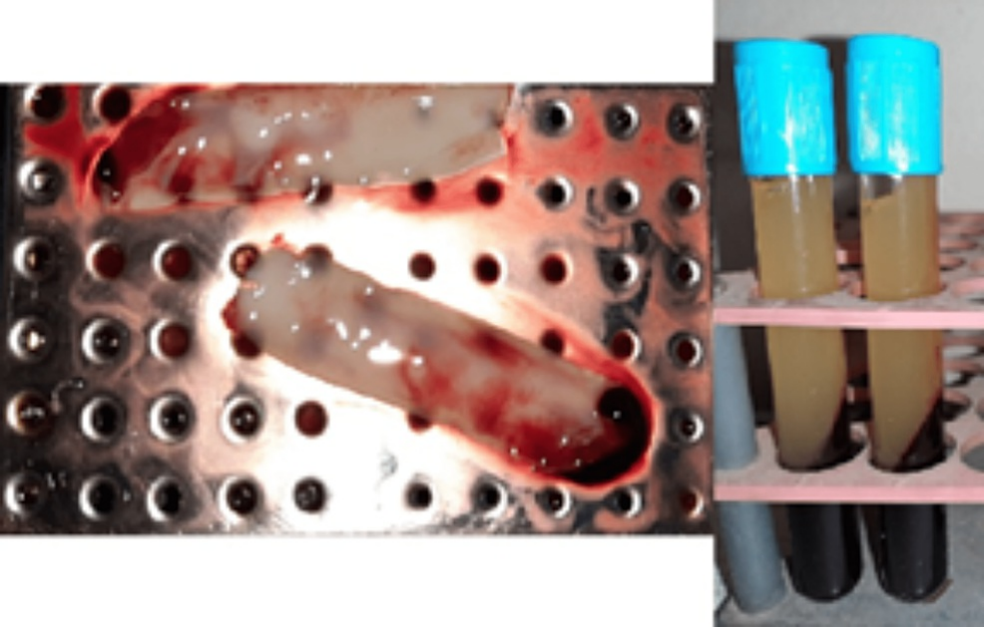
Platelet-rich fibrin leech PRF (the top layer is the leech PRF and the bottom layer is the erythrocyte layer) and the preparation of the PRF membranes

**Figure 3:**
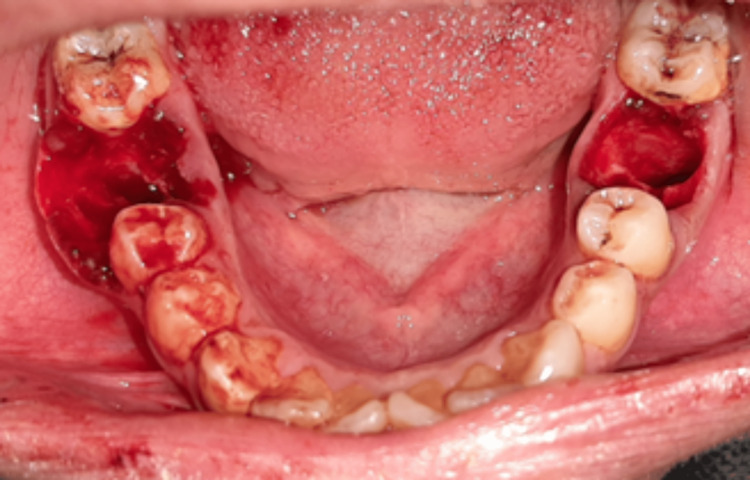
Clinical image after teeth extraction

**Figure 4:**
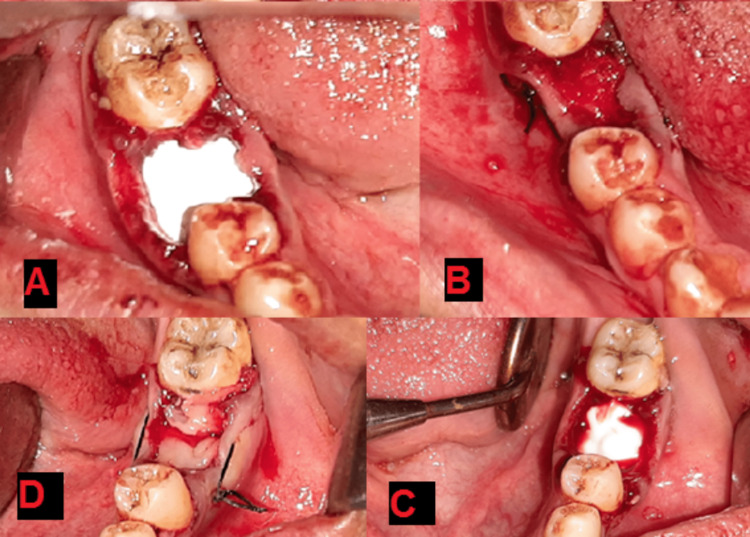
(A) The application of hydroxyapatite powder, (B) covering it with PRF membrane, and suturing procedure to fix the membrane, (C) the application of nanohydroxyapatite paste, (D) covering it with PRF membrane, and suturing to fix the membrane.

**Figure 5:**
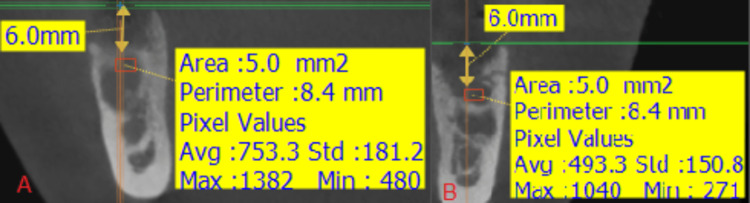
An image showing the measurement of the radiological density in the two study groups at time T0: (A) the side where the nanohydroxyapatite paste was applied, (B) the side where the hydroxyapatite powder was applied.

**Figure 6:**
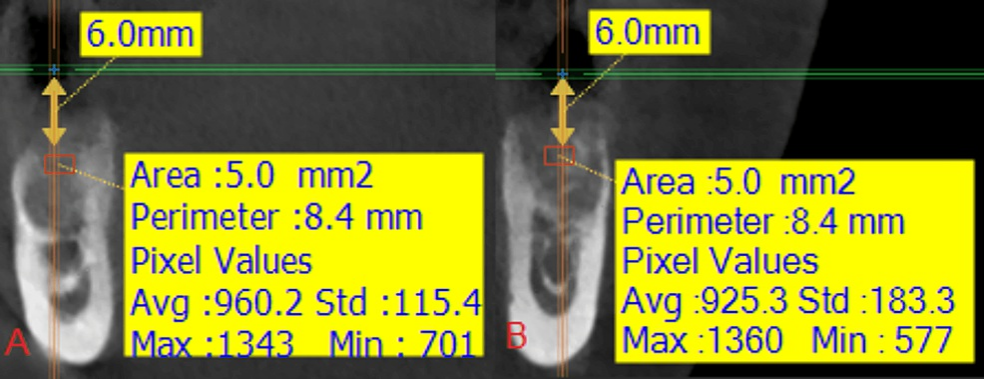
An image showing the measurement of the radiological bone density in the two study groups at time T1: (A) the side where the nanohydroxyapatite paste was applied, (B) the side where the hydroxyapatite powder was applied.

**Figure 7:**
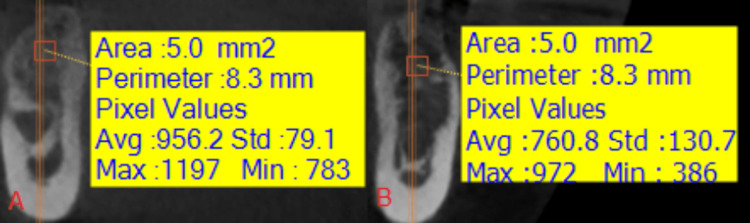
An image showing the measurement of the radiological bone density in the two study groups in time T2: (A) the side where the nanohydroxyapatite paste was applied, (B) the side where the hydroxyapatite powder was applied.

First, the intraoral clinical examination (Figure 1) and the panoramic radiographic examination (Figure 2B) were performed, and the teeth required for extraction were identified, which are the lower first molars. Then a blood sample was drawn from the patient and centrifuged to prepare the PRF membranes (Figure 2). Teeth extractions were performed (Figure 3), then bone grafting was performed with the grafts used in the research, and they were finally covered with PRF membranes (Figures 4A-4D). The radiological study was carried out in the three control periods (Figures 5-7).

 

Second Clinical Case

**Figure 8:**
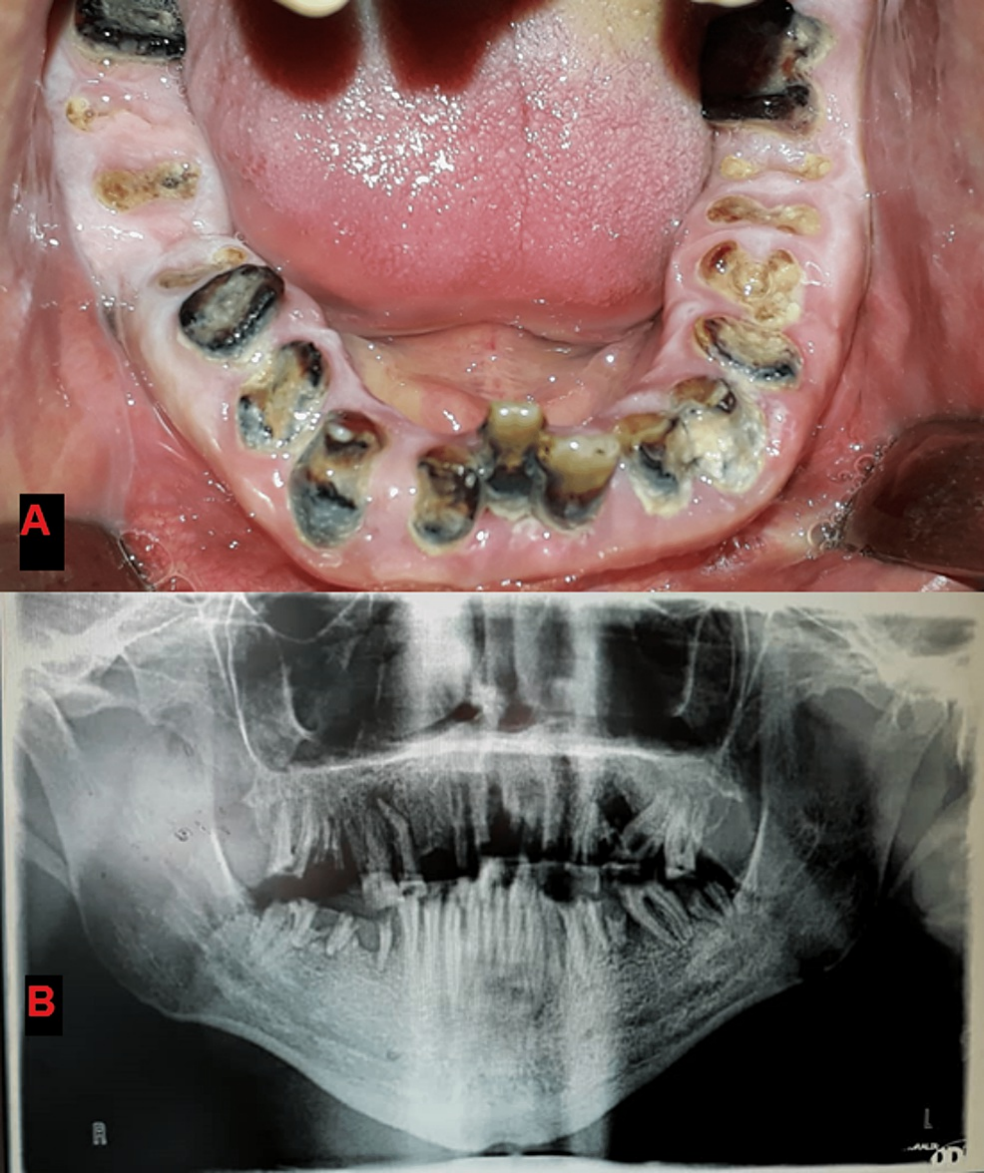
(A) Diagnostic clinical picture, (B) diagnostic panoramic image.

**Figure 9:**
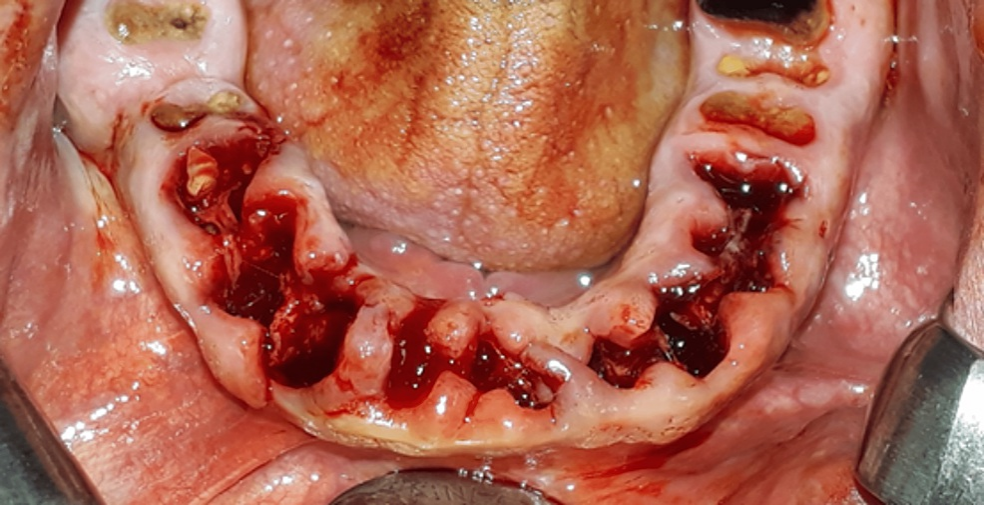
Clinical image after tooth extraction.

**Figure 10:**
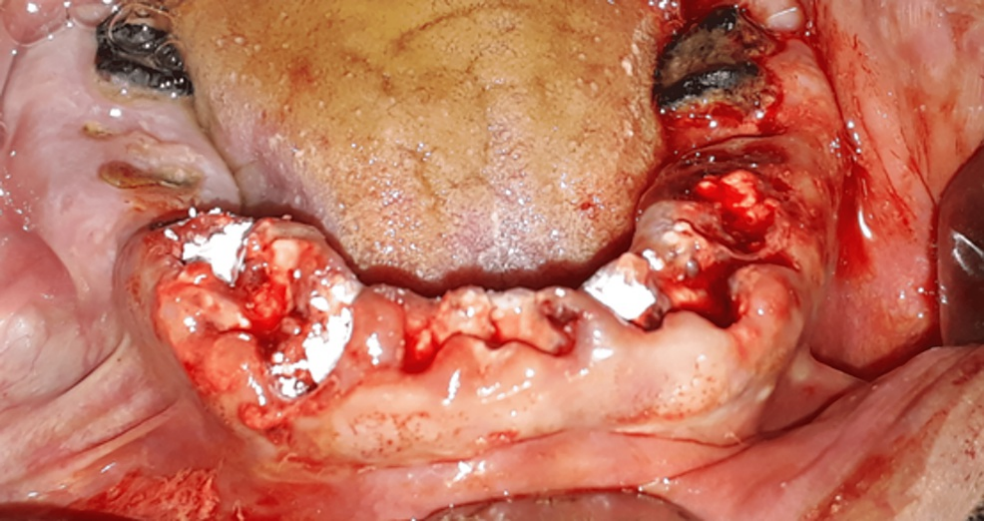
Clinical image after bone graft application.

**Figure 11:**
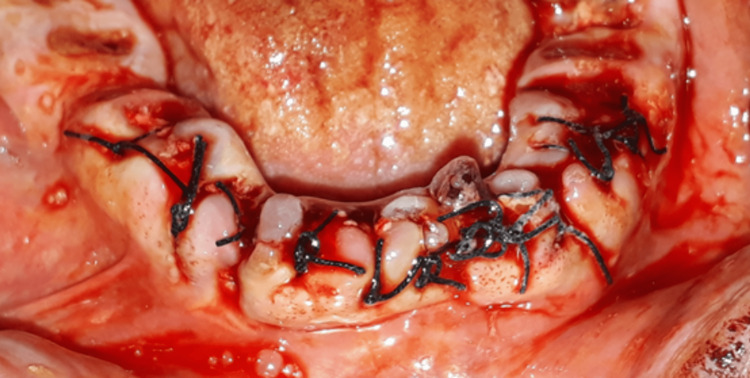
Clinical image of the extraction sites after the grafting and covering them with the PRF membrane.

**Figure 12:**
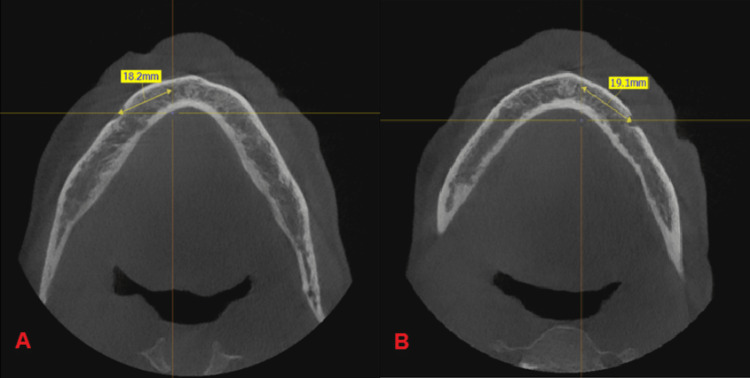
(A) Lower right quadrant reference point, (B) lower left quadrant reference point.

**Figure 13:**
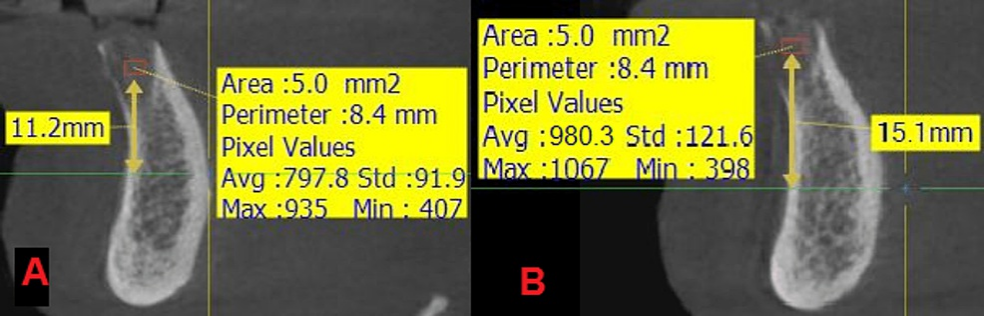
An image showing the measurement of the radiological bone density in the two study groups at time T0: (A) the side where the nanohydroxyapatite paste was applied, (B) the side where the hydroxyapatite powder was applied.

**Figure 14:**
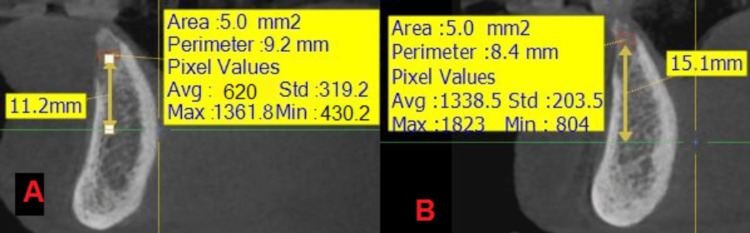
An image showing the measurement of the radiological bone density in the two study groups at time T1: (A) the side where the nanohydroxyapatite paste was applied, (B) the side where the hydroxyapatite powder was applied.

**Figure 15:**
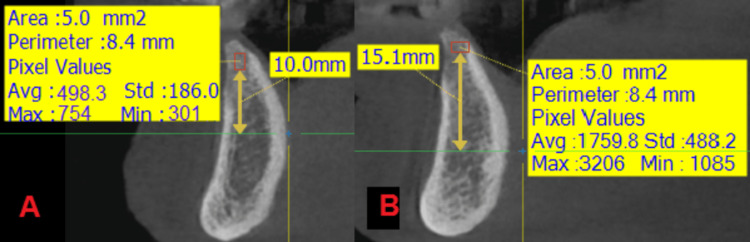
An image showing the measurement of the radiological bone density in the two study groups in time T2: (A) the side where the nanohydroxyapatite paste was applied, (B) the side where the hydroxyapatite powder was applied.

A clinical (Figure 8A) and panoramic radiographic examination (Figure 8B) was performed, and it was found that a group of symmetrical mandibular teeth had to be extracted. Extraction was performed for the required teeth (Figure 9), then the alveoli were grafted with bone grafts (Figure 10), and finally they were covered with PRF membranes and appropriate sutures were made (Figure 11). Determine the reference points for re-measurement in the same area across the three observation times (Figures 12A, 12B). The radiological study was carried out in the three control periods (Figures 13-15).

 

## Results

The study sample consisted of two groups, and the two groups together consisted of 40 alveoli. Extractions were performed for 10 patients (five males and five females) whose ages ranged between 20 and 60 years, and each of them needed an extraction for at least two teeth, symmetrical in the lower jaw. The study sample consisted of four central incisors at 10%, six lateral incisors at 15%, six canines at 15%, two first premolars at 5%, four second premolars at 10%, 10 first molars at 25%, six second molars at 15%, and two third molars by 5%.

The study sample consisted of two groups: the nanohydroxyapatite paste application group with PRF membrane and the hydroxyapatite powder application group with PRF membrane. Extraction of the indicated teeth was performed for each patient and alveolar grafting was performed using hydroxyapatite powder on one side and nanohydroxyapatite paste on the opposite side, with the two grafts covered with a PRF membrane. The mean of the radiographic bone density (RBD) in each studied group separately in three times is given in Table 1.

**Table 1:** Radiographic bone density index values for each of the two study samples.

Group	Index	Number	Minimum Value	Maximum Value	Mean	Standard Deviation
M	BD-T0	20	422.80	1265.90	824.36	277.29
	BD-T1	20	582.30	1825.00	1119.93	306.93
	BD-T2	20	750.00	1664.30	1074.14	223.62
N	BD-T0	20	264.00	988.20	629.88	193.64
	BD-T1	20	581.50	1522.60	960.67	256.88
	BD-T2	20	470.60	1100.50	743.87	180.96

The change of RBD between times T1 and T2 compared to time T0 for both group M and group N (Table 2).

**Table 2:** Change in BMD values for time T1, T2 compared with time T0 for each of the two study samples.

Group	Index	Number	Minimum Value	Maximum Value	Mean	Standard Deviation
M	BD-T1	20	-208.00	965.10	295.57	305.30
	BD-T2	20	-289.30	1132.70	249.78	385.60
N	BD-T1	20	-5.30	799.60	330.80	210.45
	BD-T2	20	-241.00	695.10	114	229.96

The change of RBD between time T2 compared to time T1 for both group M and group N (Table 3).

**Table 3:** Change of bone density between time T2 compared to time T1 for both group M and group N.

Group	Number	Minimum Value	Maximum Value	Mean	Standard Deviation
M	20	-863.20	764.80	-45.79	298.34
N	20	-540.50	381.50	-216.80	191.5

First Mauchly's test was done for assumption the of the sphericity (Table 4). One-way repeated measures ANOVA was used to test study times within the same group in each two study groups (Table 5). It has been found that (p-value > 0.05) in both groups.

**Table 4:** Mauchly's test for each of the two study groups.

Group	Mauchly's value	Approx. Chi-square	Probability value
M	.878	2.334	0.311290
N	.956	.811	0.666682

**Table 5:** One-way repeated measures ANOVA for each of the two study groups.

Group	Mean Square	F	Probability value
M	506149.946	9.178	0.000560
N	564741.440	25.317	0.0000001

Also, pairwise comparisons were conducted for both times together to study the significance of the difference in the effect of bone grafting with the two types of hydroxyapatite grafts used in the study. The study of the effect of grafting with hydroxyapatite powder showed that there were statistically significant differences when comparing the amount of change in radiographic bone density at time T0 and T1 with P < 0.05, and there were statistically significant differences when comparing the amount of change in radiographic bone density at time T0 and T2 with P < 0.05, but there were no significant differences when comparing the amount of change in radiographic bone density in T1 and T2 times with P > 0.05.

The study of the effect of grafting with nanohydroxyapatite paste showed that there were statistically significant differences when comparing the amount of change of radiographic bone density in the time T0 and T1 with P < 0.05, and there were statistically significant differences when comparing the amount of change of radiographic bone density in the time T0 and T2 with P < 0.05, and there were statistically significant differences when comparing the amount of change of radiographic bone density in the time T1 and T2 with P < 0.05 (Table 6).

**Table 6:** Comparison of the pairs of times for the radiographic bone density index for the two study groups.

Group	Index	The difference between the mean	Standard Error	Probability value
M	BD-T0/ BD-T1	-295.565	68.267	0.000361
	BD-T0/ BD-T2	-249.780	86.224	0.009242
	BD-T1/ BD-T2	45.785	66.710	0.500799
N	BD-T0/ BD-T1	-330.795	47.058	0.000001
	BD-T0/ BD-T2	-113.995	51.420	0.039022
	BD-T1/ BD-T2	216.800	42.820	0.000069

**Figure 16:**
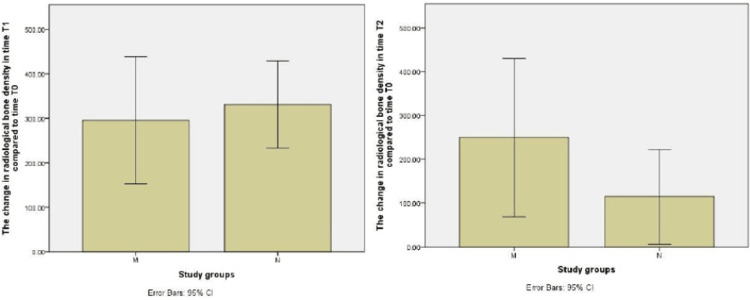
Bar charts show the change in radiological bone density in time T1 compared to time T0 (P = 0.673312) and time T2 compared with time T0 (P = 0.184194) when comparing each group M with group N.

Study of bone density change between times T1 and T2 compared to time T0 for both N and M groups using independent samples T-test is shown in Figure 16. T-test was conducted for independent samples to study the difference in radiographic bone density between times compared to time T0 for both groups of hydroxyapatite powder and nanohydroxyapatite paste, and it showed that there were no statistically significant differences (P > 0.05).

Study of the bone density difference between the times T1, T2 for each of the two groups, N and M, using T-test for independent samples

T-test was conducted for independent samples to study the radiographic bone density difference between the times T1 and T2 for both groups of hydroxyapatite powder and nanohydroxyapatite paste, and it showed that there were statistically significant differences (P < 0.05).

**Figure 17:**
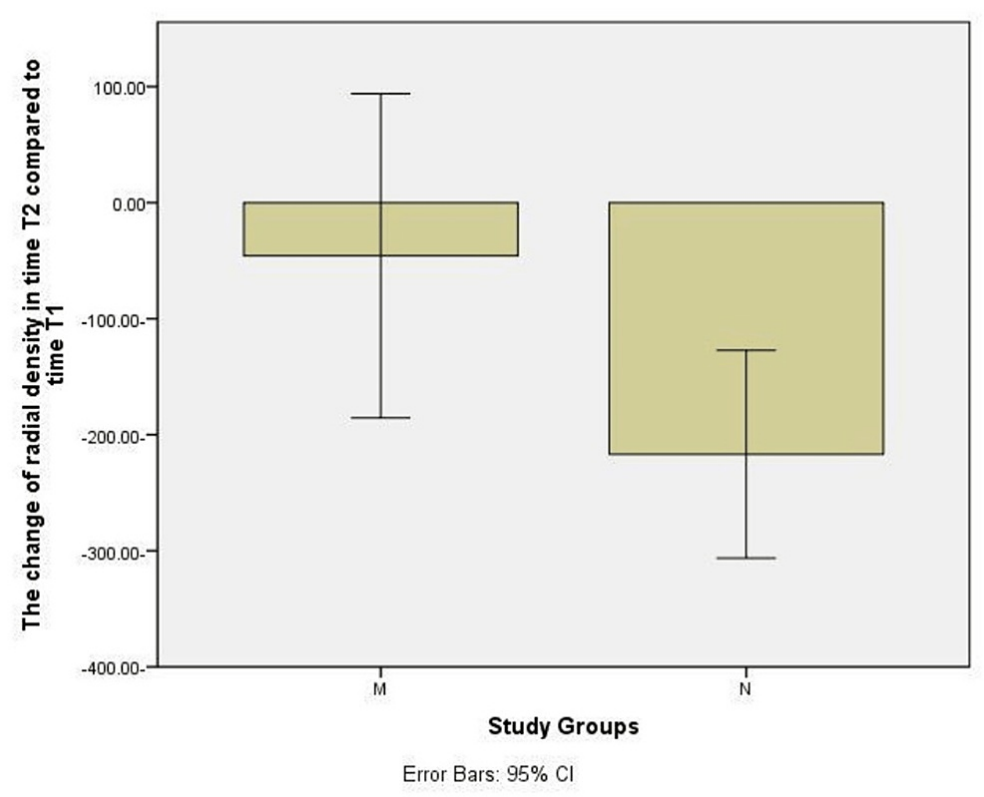
A bar chart show the change in radiological bone density in time T2 compared to time T1 when comparing group M with group N (P = 0.037366).

## Discussion

The study proved that there were statistically significant differences at the 95% confidence level in the average amount of bone resorption when using hydroxyapatite powder when comparing the amount of change in radiographic bone density between time T0 and time T1 with a value of P = 0.000, and when comparing the amount of change in radiographic bone density between time T0 and time T2 with a value of P = 0.009, and there were no statistically significant differences when comparing the amount of radiographic bone density change between time T1 and time T2 with a value of P = 0.501.

The study proved that there were statistically significant differences at the 95% confidence level in the average amount of radiographic bone density when using nanohydroxyapatite paste when comparing the amount of change in radiographic bone density between time T0 and time T1 with a value of P = 0.000, and when comparing the amount of change in radiographic bone density between time T0 and time T2 with a value of P = 0.039, and when comparing the amount of radiographic bone density change between time T1 and time T2 with a value of P = 0.000.

The statistical study also proved that there were statistically significant differences when comparing each of the two grafts used regarding the radiographic bone density between the times T1 and T2 with a value of P = 0.037 in favor of hydroxyapatite powder by retaining a greater radiographic bone density than nanohydroxyapatite paste.

We agreed with researcher Shakibaie et al. in their study on the preservation of the socket after extraction, which was based on the use of an inorganic bovine bone graft Bio-Oss, in comparison to an artificial graft consisting of hydroxyapatite with silicon dioxide that's called NanoBone, where the rate of radiographic bone density increased in a group The Bio-Oss to 399 HU with a standard deviation of ± 15.6 HU and in the NanoBone group to 699 HU with a standard deviation of ± 13.3 HU compared to the control group 352 HU with a standard deviation of ± 29.3 HU [17].

We agreed with researcher Wallace et al. in their study about the preservation of the socket after extraction, which was based on the use of decellularized dermis matrix (DDM) used as a guided bone regeneration barrier over a mineralized cancellous bone allograft (MCAB), where the radial bone density increased to a range between 385 HU and 729 HU with an average of 571 HU [18].

We disagree with the researcher Loveless et al. in their study about the preservation of the socket after extraction, which relied on the use of a freeze-dried mineralized ground cortical bone. with collagen membrane in the study group and without bone graft in the control group, the study concluded that there are no significant differences in statistical significance between the two groups, and this may be due to the difference in the type of bone graft used in the research [19]. It is recommended to do a histological study after alveolar grafting to assess the amount of bone formation.

## Conclusions

We found that the graft of hydroxyapatite powder and the nanohydroxyapatite paste increased the bone density after extraction, and there was no difference between both grafts in the time period immediately after extraction and after three months, while we found a statistically significant change when applying the nanohydroxyapatite paste in the radiological bone density after three months to six months.
